# The role of electron microscopy for the diagnosis of glomerulopathies

**DOI:** 10.1590/S1516-31802004000300006

**Published:** 2004-05-06

**Authors:** Angelo Sementilli, Luiz Antonio Moura, Marcello Fabiano Franco

**Keywords:** Electron microscopy, Kidney, Biopsy, Glomerulonephritis, Membranous glomerulonephritis, Glomerulopatia membranosa, Microscopia eletrônica, Biópsia., Glomerulonefrite

## Abstract

**CONTEXT:**

Electron microscopy has been used for the morphological diagnosis of glomerular diseases for more than three decades and its value has been widely emphasized. However, recent reports have analyzed the routine use of electron microscopy critically. Its use in other areas of diagnosis such as tumor diseases has declined considerably; in addition, in view of the unavoidable financial pressure for the reduction of costs due to investigations and diagnostic routines, the selection of cases for electron microscopy has been quite rigorous.

**OBJECTIVE:**

To identify the glomerular diseases that depend on electron microscopy for a final diagnosis, by means of reviewing renal biopsies performed over a 12-year period.

**DESIGN:**

Prospective

**SETTING:**

Hospital Ana Costa, Hospital Guilherme

Álvaro and Serviço de Anatomia Patológica de Santos, Santos, São Paulo, Brazil.

**PARTICIPANTS:**

200 consecutive renal biopsies obtained from private hospitals and the teaching hospital from 1979 to 1991 were studied.

**MAIN MEASUREMENTS:**

All cases were analyzed via light microscopy, immunofluorescence and electron microscopy. The diagnosis was first made via light microscopy plus immunofluorescence and then via electron microscopy.

**RESULTS:**

Electron microscopy was diagnostic or essential for diagnosis in 10.0% of the cases, corresponding to 3.4% of primary glomerulopathies and 100% of hereditary glomerulopathies. Electron microscopy was contributory (useful) to the diagnosis in 5.5% of the cases, confirming the preliminary diagnosis formulated on the basis of clinical and laboratory data and light microscopy plus immunofluorescence findings. We obtained a 7.5% rate of discordant immunofluorescence, which was considered as such when negative immunofluorescence findings were not confirmed by electron microscopy. The final diagnosis with the use of light microscopy plus immunofluorescence alone was 77.0%.

**CONCLUSIONS:**

It was possible to diagnose with certainty a great percentage of glomerulopathies (82.5-90% of the cases) based on the light microscopy and immunofluorescence findings alone. Electron microscopy was essential for the diagnosis of hereditary nephropathies.

## RESUMO

**CONTEXTO:**

A microscopia eletrônica tem sido usada há mais de três décadas para o diagnóstico morfológico das doenças glomerulares e seu valor tem sido amplamente enfatizado. Entretanto, relatos recentes têm analisado criticamente o uso rotineiro da microscopia eletrônica. O seu uso em outras áreas de diagnóstico como doenças tumorais tem declinado consideravelmente. Além disso, em virtude da inevitável pressão financeira para redução dos custos da investigação na rotina diagnóstica, a seleção dos casos para microscopia eletrônica tem sido rigorosa.

**OBJETIVO:**

Com o intuito de se identificarem as doenças glomerulares que dependem da microscopia eletrônica para o diagnóstico final, foram revisadas biópsias renais recebidas no período de 12 anos.

**TIPO DE ESTUDO:**

Prospectivo

**LOCAL:**

Hospital Ana Costa, Hospital Guilherme Álvaro e Serviço de Anatomia Patológica de Santos, São Paulo, Brasil.

**PARTICIPANTES:**

200 biópsias renais consecutivas, obtidas de hospital privado e hospitalescola de 1979 a 1991.

**PRINCIPAIS VARIÁVEIS:**

Todos os casos foram analisados por microscopia óptica, imunofluorescência e microscopia eletrônica. O diagnóstico foi inicialmente feito por microscopia óptica e de imunofluorescência, e posteriormente pela microscopia eletrônica.

**RESULTADOS:**

A microscopia eletrônica foi diagnóstica ou essencial para o diagnóstico em 10,0% dos casos, correspondendo a 3,4% de glomerulopatias primárias e 100% das glomerulopatias hereditárias. A microscopia eletrônica foi contributiva para o diagnóstico em 5,5% dos casos, confirmando os diagnósticos formulados com base em dados clínicos e laboratoriais, e achados de microscopia óptica e de imunofluorescência. Obtivemos 7,5% de imunofluorescências discordantes, assim consideradas quando os achados de imunofluorescência não foram confirmados pela microscopia eletrônica. Em 77,0% dos casos, o diagnóstico final pôde ser estabelecido exclusivamente com base nos achados de microscopia óptica e de imunofluorescência.

**CONCLUSÕES:**

Foi possível diagnosticar com exatidão grande porcentagem (82,5 - 90,0%) dos casos com base nos achados isolados de microscopia óptica e de imunofluorescência. A microscopia eletrônica foi essencial para o diagnóstico das nefropatias hereditárias.

## INTRODUCTION

Electron microscopy has been used for the morphological diagnosis of glomerular diseases for more than three decades and its value has been widely emphasized.^1^ However, recent reports have analyzed the routine use of electron microscopy critically. Its use in other areas of diagnosis such as tumor diseases has declined considerably; in addition, in view of the unavoidable financial pressure for the reduction of costs due to investigations and diagnostic routines, the selection of cases for electron microscopy has been quite rigorous.^2-4^

Some investigators have observed that about 85% of renal biopsies had an indication of electron microscopy for diagnostic confirmation.^5^ Routine diagnostic electron microscopy has proved to be of high value in the differential diagnosis of nephrotic syndrome, especially in early membranous glomerulonephritis and in cases of minimal lesion glomerulopathy. The use of electron microscopy for the classification of glomerular diseases has been well established, and the technique can also be used for therapeutic monitoring.^6^

Some lesions detected via light microscopy and immunofluorescence can be better characterized by electron microscopy, as is the case for the localization of immune deposits and structural changes in the glomerular basement membrane. The use of electron microscopy has allowed the recognition of changes not observed under light microscopy, thereby contributing to the understanding of the pathogenesis of renal diseases.^7^ This is the technique used for the determination of glomerular basement membrane damage in non-immune glomerulopathies such as Alport syndrome, thin basement membrane disease and nephrotic syndrome with minimal lesion glomerulopathy.^8^

In thin basement membrane disease, light microscopy only reveals the presence of blood casts in the tubular lumen. The early thickening of the glomerular basement membrane, which may occur in diabetic nephropathy, hypertension and glomerulonephritis, can also be seen only via electron microscopy. The localization of immunocomplexes is important for defining the type of glomerulonephritis. Finally, ultrastructural evaluation is essential for adequate characterization of fibrillar glomerulonephritis such as microfibrillar and immunotactoid glomerulonephritis.^9,10^

The objective of the present study was to analyze the role of electron microscopy examination for the diagnosis of glomerular disease in a consecutive series of biopsies analyzed by the same pathologist with the systematic use of light microscopy, immunofluorescence and electron microscopy.

## METHODS

A total of 200 consecutive renal biopsies obtained from private hospitals and the teaching hospital of the Santos School of Medical Sciences, State of São Paulo, from 1979 to 1991 were studied via light microscopy, immunofluorescence and electron microscopy. Most of the biopsies were obtained using a needle and about 15% were obtained via open surgery, especially the biopsies from children.

The renal fragments were received for analysis without prior fixing. They were divided into three portions: I) the extremities were fixed in glutaraldehyde and reserved for electron microscopy; II) the central portion was frozen at −20° C and used for immunofluorescence; and III) the remaining portions were fixed in 10% formalin for paraffin sections and examination via light microscopy. The material was processed for electron microscopy by resin embedding and cutting into 750 Å sections using a diamond knife. The sections were placed on a net for observation under a Philips electron microscope.

Immunofluorescence involved the search for immunoglobulins A, G and M (IgA, IgG and IgM), C3 and C1q complement components, and also albumin and fibrinogen. Sections of 3 to 4 μm were obtained using a cryostat and fixed in cold acetone. After incubation and washing in phosphate-buffered saline solution (PBS, pH 7.2), readings were taken using a Zeiss fluorescence microscope. For light microscopy analysis, the paraffin blocks were cut into 3 to 5 μm sections, which were stained using hematoxylin-eosin, Masson trichrome, periodic acid Schiff, and silver impregnation.

The light microscopy, immunofluorescence and electron microscopy findings as a whole were reviewed by two pathologists for definition of the final diagnosis. Each case was first analyzed using light microscopy and immunofluorescence findings together with the clinical and laboratory data for the morphological and nosological interpretation of the glomerulopathy. These findings were then reevaluated together with the ultrastructural study in order to determine the impact of electron microscopy on the diagnosis of the glomerular disease.

## RESULTS

The distribution of the 200 cases studied according to the World Health Organization classification of glomerulonephritis^11^ is presented in Table 1. Primary glomerulonephritis was observed in 59.5% of the cases, systemic diseases with glomerulonephritis in 24%, vascular disease with glomerulonephritis in 7.0%, metabolic disease with glomerulonephritis in 1.0%, hereditary nephropathies in 7.5%, and diverse glomerular diseases in 1.0%. There was sharp predominance of minimal lesion glomerulopathy in the primary glomerulonephritis group and of lupus nephritis in the glomerulonephritis associated with systemic diseases.

**Table 1:** Distribution of 200 cases of glomerulopathies according to the 1995 World Health Organization classification^11^

	No. cases	%
**I- Primary glomerulopathies**		
Minimal lesions	32	16.0
Focal and segmental glomerulosclerosis	13	6.5
Diffuse glomerulonephritis		
Membranous glomerulonephritis	14	7.0
Proliferative glomerulonephritis		
Mesangial proliferative glomerulonephritis	17	8.5
Endocapillary proliferative glomerulonephritis	19	9.5
Mesangiocapillary glomerulonephritis type 1 and 3	21	10.5
Crescentic glomerulonephritis (extra-capillary)	2	1.0
Unclassified glomerulonephritis	1	0.5
**Partial Total**	**119**	**59.5**
**II- Glomerulopathies associated with systemic diseases**		
Lupus nephritis	35	17.5
IgA nephropathy (Berger's disease)	13	6.5
**Partial Total**	**48**	**24.0**
**III- Glomerulopathies associated with vascular disorders**		
Nodosa polyarteritis	1	0.5
Hemolytic uremic syndrome	2	1.0
Benign nephrosclerosis	11	5.5
**Partial Total**	**14**	**7.0**
**IV- Glomerulopathies associated with metabolic diseases**		
Diabetic glomerulosclerosis	1	0.5
Amyloidosis	1	0.5
**Partial Total**	**2**	**1.0**
**V- Hereditary nephropathies**		
Alport syndrome	1	0.5
Recurrent hematuria with normal glomerular basement membrane	11	5.5
Thin basement membrane disease	2	1.0
Congenital nephrotic syndrome	1	0.5
**Partial Total**	**15**	**7.5**
**VI- Glomerulopathies, miscellaneous**		
Eclampsia nephropathy	1	0.5
Acute interstitial nephritis	1	0.5
Partial Total	2	1.0
**Total**	**200**	**100**

The case series was evaluated according to: 1) number of cases in which the diagnosis was reached via light microscopy plus immunofluorescence alone; 2) number of cases in which immunofluorescence was considered discordant from the electron microscopy findings; and 3) number of cases in which electron microscopy was interpreted as essential for the diagnosis (Table 2).

**Table 2:** Contribution of different diagnostic methods in 200 cases of glomerulopathies

		Light microscopy plus immunofluorescence	Discrepant ímmunofluorescence microscopy	Useful electron microscopy	Essential electron microscopy	Total
**I-Primary glomerulopathies**						
Minimal lesion		29	2	1	-	32
Focal and segmental glomerulosclerosis		12	-	1	-	13
Membranous glomerulonephritis		9	3	2	-	14
Mesangial proliferative glomerulonephritis		15	1	1	-	17
Endocapillary proliferative glomerulonephritis		13	3	2	1	19
Mesangiocapillary glomerulonephritis (types 1 and 3)		16	2	-	3	21
Crescentic glomerulonephritis (extra-capillary)		1	-	1	-	2
Unclassified glomerulonephritis		1	-	-	-	1
**II-Secondary glomerulopathies**						
A) Systemic diseases						
Lupus nephritis		33	2	-	-	35
Berger disease		8	2	3	-	13
B) Vascular diseases						
Nodosa polyarteritis		1	-	-	-	1
Hemolytic uremic syndrome		2	-	-	-	2
Benign nephrosclerosis		11	-	-	-	11
C) Metabolic diseases						
Diabetic glomerulosclerosis		1	-	-	-	1
Amyloidosis		1	-	-	-	1
**III-Hereditary nephropathies**						
Alport syndrome		-	-	-	1	1
Recurrent hematuria with normal glomerular basement membrane		-	-	-	11	11
Thin basement membrane disease		-	-	-	2	2
Congenital nephrotic syndrome		-	-	-	1	1
**IV-Glomerulopathies, miscellaneous**						
Eclampsia nephropathy		1	-	-	-	1
Acute interstitial nephritis		-	-	-	1	1
	**Total**	**154**	**15**	**11**	**20**	**200**
	**%**	**77.0%**	**7.5%**	**5.5%**	**10.0%**	**100%**

In 154 biopsies (77.0%), the morphological diagnosis of glomerular disease was based on light microscopy and immunofluorescence findings alone. In 15 cases (7.5%), immunofluorescence yielded results that were not confirmed by electron microscopy (discrepant immunofluorescence). In 11 cases (5.5%), electron microscopy confirmed the light microscopy and fluorescence findings (contributory electron microscopy). In 20 cases (10.0%), the electron microscopy was essential for the final diagnosis.

Electron microscopy was mainly contributory or confirmatory for diagnosis when it revealed or confirmed important morphological elements that had not been clearly observed via light microscopy or immunofluorescence, as follows: cases of membranous glomerulonephritis in the early state with negative immunofluorescence, in which electron microscopy revealed electron-dense subepithelial deposits; cases of proliferative endocapillary glomerulonephritis with negative immunofluorescence in which electron microscopy revealed electron-dense subepithelial “hump” type deposits; cases of IgA nephropathy with doubtful immunofluorescence examination, in which electron microscopy revealed electron-dense paramesangial deposits.

In the four cases of proliferative glomerulonephritis (one case of endocapillary proliferative glomerulonephritis and three cases of mesangiocapillary glomerulonephritis), electron microscopy was diagnostic by demonstrating subepithelial and subendothelial electron-dense deposits, respectively.

Within the group in which electron microscopy was essential to the diagnosis, the highest percentage of cases consisted of hereditary nephropathies (15 cases), divided into Alport syndrome^1^, benign recurrent hematuria^11^, thin basement membrane disease^2^, and congenital nephrotic syndrome^1^.

## DISCUSSION

Although renal biopsies started to be performed in medical practice in 1951, pioneered by Iversen & Brun,^12^ it was only 20 years later that the importance of immunofluorescence became emphasized in relation to complementary assessment of the anatomopathological evaluation.

Subsequently, Habib & Gluber,^13^ in 1983, tried to correlate light microscopy and electron microscopy findings and define the pathogenesis via immunofluorescence. No attempt was made in any of these reports to systematically quantify the importance of each procedure or determine what conditions would not be diagnosed in the absence of one of the stains or methods used.

The importance of such assessment resides in the fact that most histopathology laboratories in Brazil do not have immunofluorescence capability, and only a minority can use electron microscopy, which is usually performed in the laboratories of the major university hospitals. For countries with fewer resources, the diagnosis of glomerular disease needs to be made possible by first using less expensive methods before employing electron microscopy.^2-4^

In the presence of minimal lesion glomerulopathy, although the primary lesion is ultrastructural, with podocyte effacement, normal light microscopy and negative immunofluorescence findings in combination with clinical data are indicative for the diagnosis. In focal and segmental glomerulosclerosis, immunofluorescence can reveal trapping of IgM and/or C3 in the sclerosed glomeruli, and thus electron microscopy is important for the diagnosis since it reveals fusion of the foot processes in the glomeruli, which appear normal via light microscopy and without electron-dense deposits. In membranous glomerulonephritis, the diagnosis may be difficult in stage I of the disease since, at the beginning, the changes are not evident via special staining, with the absence of spikes in silver impregnation.

Some diagnosis can be made only via immunofluorescence, but when this procedure is considered erroneously negative, as in cases of weak or irregular staining due to previous treatment or technical error, the final diagnosis will depend on electron microscopy examination finding electron-dense deposits.^9^

Endocapillary proliferative glomerulonephritis usually presents no diagnostic difficulties when it shows endocapillary proliferation and neutrophilic exudation via light microscopy and granular deposits predominantly of C3 via immunofluorescence. If immunofluorescence is inconclusive, it is important to make a differential diagnosis with other entities in which complement activation occurs and neutrophils are present. In such cases, the differential diagnosis is made via electron microscopy when epimembranous deposits with a “hump” pattern are detected. Mesangiocapillary glomerulonephritis is frequently recognized by glomerular basement membrane duplication, which is well demonstrated in most cases by silver staining. In immunofluorescence, the detection of granular and peripheral C3 deposits contributes to the diagnosis.

Only three cases in the present series were defined by electron microscopy. One case had glomerular changes similar to mesangio-capillary glomerulonephritis, in which fibrillar deposits were detected via electron microscopy. The other two cases were in the early phases of mesangiocapillary glomerulonephritis, with the detection of submembranous deposits via electron microscopy. Such ultrastructural finding allowed differentiation between mesangiocapillary glomerulonephritis and acute diffuse glomerulonephritis, which would not have occurred if only light microscopy and immunofluorescence had been used. None of our cases was assigned to mesangiocapillary glomerulonephritis type II.

Among the systemic diseases, lupus nephritis was the most frequent. Light microscopy and immunofluorescence are sufficient for adequate definition of the various types of lupus lesion and for identifying active or chronic lesion.^14,15^ In Berger's disease, light microscopy can have various presentations, ranging from normal glomeruli to glomeruli with focal lesion or global sclerosis. The definitive diagnosis of IgA nephropathy is obtained via immunofluorescence with the detection of IgA of mesangial location. Electron microscopy is complementary, revealing electron-dense deposits in paramesangial regions. Although not specific, such findings strongly support the diagnosis of IgA nephropathy.

When immunofluorescence does not demonstrate these deposits, the electron microscopy findings can be indicative of the entity, especially in the presence of predominant hematuria. In the group of metabolic and vascular diseases, the diagnostic conclusion is normally reached via light microscopy, since immunofluorescence is negative.^16^ In fibrillar glomerulonephritis, special stains such as Congo red for the demonstration of amyloid are useful for the diagnostic procedures, except in the early stage. None of our cases showed scarce amyloid deposits via electron microscopy, which would not have shown positivity using Congo red staining. In nonamyloid cases, examination via electron microscopy and other clinical and laboratory data are essential for the diagnosis.

In the hereditary nephropathies group, electron microscopy makes the definitive diagnosis. Light microscopy can be normal at first and immunofluorescence is always negative, but electron microscopy allows documentation of alterations at the glomerular basement membrane level. When the glomerular basement membrane is delaminated and of variable thickness, Alport syndrome is characterized. When the membrane thickness is very reduced, usually to about one-third of normal or approximately 200 nm, thin basement membrane disease is characterized.^17^

When nephrotic syndrome is observed in newborn infants and the clinical data is reminiscent of minimal lesion glomerulopathy, congenital nephrotic syndrome is diagnosed. Nephritic cases with normal findings from light microscopy, immunofluorescence and electron microscopy are classified as recurrent hematuria with normal glomerular basement membrane. A large part of our group was assigned to this category because recurrent hematuria is a clinical indication for biopsy, and this is useful for ruling out more severe disease of poor prognosis.

With regard to the two cases of diverse glomerular diseases, light microscopy defined the case of eclampsia nephropathy and electron microscopy was essential for the diagnosis in the case of acute interstitial nephritis, in order to rule out other conditions accompanied by hematuria, such as hereditary nephropathy.

A survey of the literature showed that there is no publication studying the impact and cost/benefit relationship of electron microscopy in the diagnosis of glomerulopathies in routine service, among consecutive renal biopsies. Most studies only mention the generic importance of its use or its importance for groups of glomerulopathies, as can be seen in Table 3.

**Table 3:** List of publications emphasizing the use of electron microscopy for the diagnosis of glomerulopathies

Study and date	No of cases	Findings/conclusions
Muehrcke et al., 1969^18^	179 cases	In 6% of the cases, electron microscopy contributed to the diagnosis. Renal biopsy reserved for rare cases.
Tighe & Jones., 1970^5^	100 cases	Great value of electron microscopy in differential diagnosis of nephrotic syndrome. Limitation of the method due to the high cost and long time required.
Siegel et al., 1973^19^	213 cases	In 48% of the cases, electron microscopy contributed to the diagnosis and conduct. One electron microscopy case differed from light microscopy. Since light microscopy does not allow prediction of when to use electron microscopy, the method should be used routinely.
Ben-Bassat et al., 1974^20^	37 cases	Great usefulness in nephrotic syndrome for the differential diagnosis of minimal lesion and early membranous glomerulonephritis.
Spargo., 1975^1^	review of literature	Practical uses of electron microscopy for the diagnosis of glomerular disease. Elec-tron microscopy to be used whenever a biopsy is assessed critically.
Dische & Parsons., 1977^21^	134 cases	Assessment of the contribution of immunofluorescence and electron microscopy. For a precise diagnosis, it is essential to complement light microscopy, preferably with immunofluorescence and electron microscopy.
Collan et al., 1978^22^	28 cases	Retrospective study in cases with clinical suspicion and symptoms of glomerular disease. Electron microscopy significantly contributed to the diagnosis by demon strating immune deposits.
Skjorten & Halvorsen, 1981^23^	200 cases	Electron microscopy was useful in 45% of glomerulonephritis and changed the diagnosis in 34%. Electron microscopy should be routinely used when glomeru lonephritis is suspected.
Pearson et al., 1994^6^	88 cases	Electron microscopy used together with light microscopy and immunofluorescence. Electron microscopy was useful in 50%, essential in 25% and of no use in 25%. It is mainly useful for minimal lesion and related entities.

Pearson et al.,^6^ in 1994, concluded that electron microscopy plays an important role in the diagnosis of renal disease and therefore renal tissue should be submitted to electron microscopy whenever possible. In some selected cases, when light microscopy and immunofluorescence results are already known, the ultrastructural examination could be predicted. Electron microscopy would be particularly useful for the differential diagnosis of glomerulopathies that progress with nephrotic syndrome.

In our series, electron microscopy was essential for the diagnosis in 10% of the cases, i.e. the correct diagnosis would not have been possible without it. It was contributory in 5.5% of the cases, in situations in which it confirmed the light microscopy plus immunofluorescence findings. If we succeed in reducing the percentage of immunofluorescence that is considered discrepant (7.5%), by means of improved controls, reduction of technical errors and avoiding post-treatment biopsies (false-negative results), we would reach 90% accuracy in the diagnosis of biopsied glomerulopathies, using only light microscopy plus immunofluorescence.

## CONCLUSION

1. The frequencies of glomerulopathies diagnosed by biopsy, in the city of Santos, Brazil, not including cases of renal transplantation, were as follows: predominance of primary glomerulopathies (59.5%); lower frequency for glomerulopathies associated with systemic diseases (24%); similar low frequencies for glomerulopathies associated with vascular diseases (7%) and hereditary nephropathies (7.5%); very low frequency for glomerulopathies associated with metabolic diseases (1.0%) and miscellaneous (1.0%).
2. It was possible to diagnose with certainty a large percentage of the glomerulopathies (82.5 to 90% of the cases) based on the light microscopy and immunofluorescence findings alone. Electron microscopy was essential for the diagnosis of hereditary nephropathies.
